# Addressing ocean planning challenges in a highly crowded sea space: a case study for the regional sea of Catalonia (Western Mediterranean)

**DOI:** 10.12688/openreseurope.16836.1

**Published:** 2024-03-01

**Authors:** Daniel Depellegrin, Stefano Menegon, Andrej Abramic, Simón Aguado Hernandez, Francesca Larosa, Santiago Salvador, Carolina Marti Llambrich

**Affiliations:** 1 Department of Geography, University of Girona, Girona, Catalonia, 17004, Spain; 2 Institute of Marine Sciences, National Research Council, ISMAR-CNR, Venice, Italy; 3 Scientific & Technological Marine Park, University Las Palmas de Gran Canaria, Biodiversity & Conservation Research Group, Institute of Sustainable Aquaculture and Marine Ecosystems, IU-ECOAQUA, Telde, Spain; 4 Grupo de Investigación de Economía, Territorio y Medio Ambiente, Universidad Politécnica de Cartagena, Murcia, Spain; 5 Faculty of Business And Communication Studies, Universidad Internacional de La Rioja, UNIR, Logroño, Spain; 6 Euro-Mediterranean Center on Climate Change, Venice, Italy; 7 KTH Climate Action Centre, KTH Royal Institute of Technology, Stockholm, Sweden; 8 Ephyslab – Environmental Physics Laboratory, University of Vigo, Vigo, Spain; 9 Department of Public Law, University of Vigo, Vigo, Spain

**Keywords:** Maritime Spatial Planning, spatial conflicts, MSFD pressures, stressors, offshore wind energy, aquaculture, marine protection, Spain

## Abstract

**Background:**

This study performs an exploratory analysis of current-future sustainability challenges for ocean planning for the regional seas of Catalonia located in the Western Mediterranean (Spain).

**Methods:**

To address the challenges we develop an Maritime Spatial Planning (MSP)-oriented geodatabase of maritime activities and deploy three spatial models: 1) an analysis of regional contribution to the 30% protection commitment with Biodiversity Strategy 2030; 2) a spatial Maritime Use Conflict (MUC) analysis to address current and future maritime activities interactions and 3) the StressorGenerator QGIS application to locate current and anticipate future sea areas of highest anthropogenic stress.

**Results & Conclusions:**

Results show that the i) study area is one of the most protected sea areas in the Mediterranean (44–51% of sea space protected); ii) anthropogenic stressors are highest in 1–4 nautical miles coastal areas, where maritime activities agglomerate, in the Gulf of Roses and Gulf of Saint Jordi. iii) According to the available datasets commercial fishery is causing highest conflict score inside protected areas. Potential new aquaculture sites are causing highest conflict in Internal Waters and the high potential areas for energy cause comparably low to negligible spatial conflicts with other uses. We discuss the added value of performing regional MSP exercises and define five challenges for regional ocean sustainability, namely: Marine protection beyond percentage, offshore wind energy: a new space demand, crowded coastal areas, multi-level governance of the regional sea and MSP knowledge gaps.

## 1. Introduction

The coastal region of Catalonia (Spain, Western Mediterranean) belongs to the Mediterranean regions with highest revenue in relation to Blue Economy ( BE, 2020). The Blue Economy is estimated to have directly employed 214496 people in 2017 (5.8% of the total employment in Catalonia), generated €25 billion of revenue and €7.3 billion of Gross Value Added (GVA) (3.4 % of the GVA in Catalonia; BE, 2020). In addition, the regional authorities have launched a regional Blue Economy Strategy of Catalonia 2030 ( Estratègia Marítima, 2021) with ambitions for sustainable development across Blue Economy sectors and the marine resource. In 2017, through a Royal Decree 363/2017, the Spanish government transposed to Spanish law the European Maritime Spatial Planning (MSP) Directive 2014/89/EU, setting the path to a national framework for maritime spatial management. This resulted in 2021 to the drafting of the first maritime spatial plan of Spain, hereafter POEM - “ *Planes de Ordenación del Espacio Maritime*” ( POEM, 2021). The process is preceded by an increasing segment of scientific literature in Spain, applying analysis techniques to address ecosystem-based management and the spatial organization of present and future Blue Economy activities. This includes among others the analysis of future sectorial space demands for marine renewable energy ( Abramic *et al.*, 2021; Díaz & Soares, 2021; Pınarbaşı *et al.*, 2019; Salvador *et al.*, 2019), transboundary challenges in MSP ( Gómez-Ballesteros, *et al.*, 2021; Pınarbaşı *et al.*, 2020) environmental impact assessment of maritime activities ( Abramic *et al.*, 2022; Muñoz *et al.*, 2018) or potentials for ocean multi-use ( Saenz-Aguirre *et al.*, 2022; Veigas & Iglesias, 2013). The majority of these studies focus on study areas at regional or at spatial subdivision scale. At the current stage no studies exist, addressing marine multi-sectoral interactions, nor do regional ocean planning studies exist for Catalonia region addressing existing and future MSP challenges for the region. According to a review of MSP practices around Europe performed by van den Burg *et al.* (2023), 24% of MSP studies have a regional geographic scale. In fact regional MSP case study funded by European and national funding entities resulted into valuable contribution to the national MSP processes in different regions of European seas: examples include the Scottish Sustainable Marine Environment Initiative ( SSMEI, 2018) aimed at testing new approaches to improve sustainable management of Scottish marine resources through the setup of pilot projects (e.g. Shucksmith *et al.*, 2014), the ICZM-MSP regional pilot study in Emilia-Romagna Region ( Barbanti & Perini, 2018; Farella *et al.*, 2020) or the case study on assessing and mapping marine ecosystem services in the Latvian MSP through the ESMERALDA Project ( Burkhard *et al.*, 2018; Veidemane *et al.*, 2017). Given these experiences and the iterative character of ocean planning ( Ehler & Douvere, 2009), it is of pivotal importance now and in future to perform regional ocean planning exercises that can foster knowledge and competences in MSP and inform decision-makers and Blue Economy stakeholders on the regional challenges of sustainable use of maritime space. This research applies MSP-driven geospatial analysis techniques to 1) quantify regional contribution to EU Biodiversity Target 2030 of 30% sea protection; 2) locate interactions in terms of spatial conflict analysis among present and future maritime sectors and 3) applies anthropogenic stress areas in the light of the POEM’s zoning typologies.

Based on the results we formulate five emerging regional challenges for sustainable use of the maritime space, suggest actionable (non-) spatial management measures and highlight the benefits and challenges of regional MSP research.

## 2. Methods

**Figure 1.**
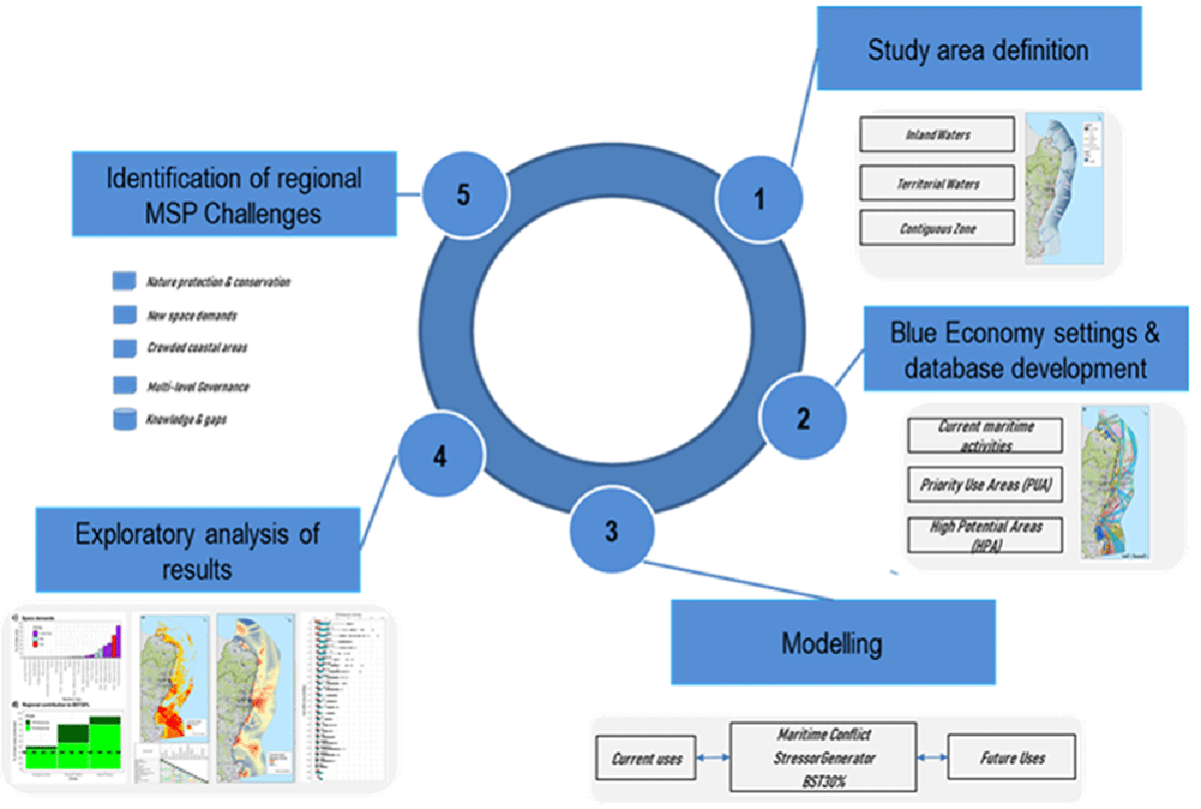
Conceptual framework applied in this case study area.

The exploratory analysis is based on a step-wise approach highlighted in the conceptual framework below ( Figure 1). In summary this includes: 1) the definition of the study area into three zoning areas (see section 2.1), 2) the creation of a multi-sectoral database of the most representative Blue Economy activities in Catalonia region (see Annex 1 for Blue Economy characterization in Depellegrin & Martí Llambrich, 2024) including the human activities emerging from the zoning solutions settled in the Strategic Environmental Assessment (SEA) of the POEM; 3) the analysis of regional contribution of Biodiversity Strategy 2030 (see section 2.4); the application of a maritime use conflict (MUC) analysis using the Tools4MSP Modelling Framework (Tools4MSP, 2022; section 2.5); application of the QGIS-based *StressorGenetor* Plugin ( section 2.6); 4) Exploratory analysis and evaluation of results oriented to address the regional challenges on sustainable use of maritime space (see section 3 and 4) and the relevance of the applied regional case studies for further research in the area (see section 5); and 5) identification of emerging challenges in sustainable use of maritime space.

### 2.1. Study area definition

**Figure 2.**
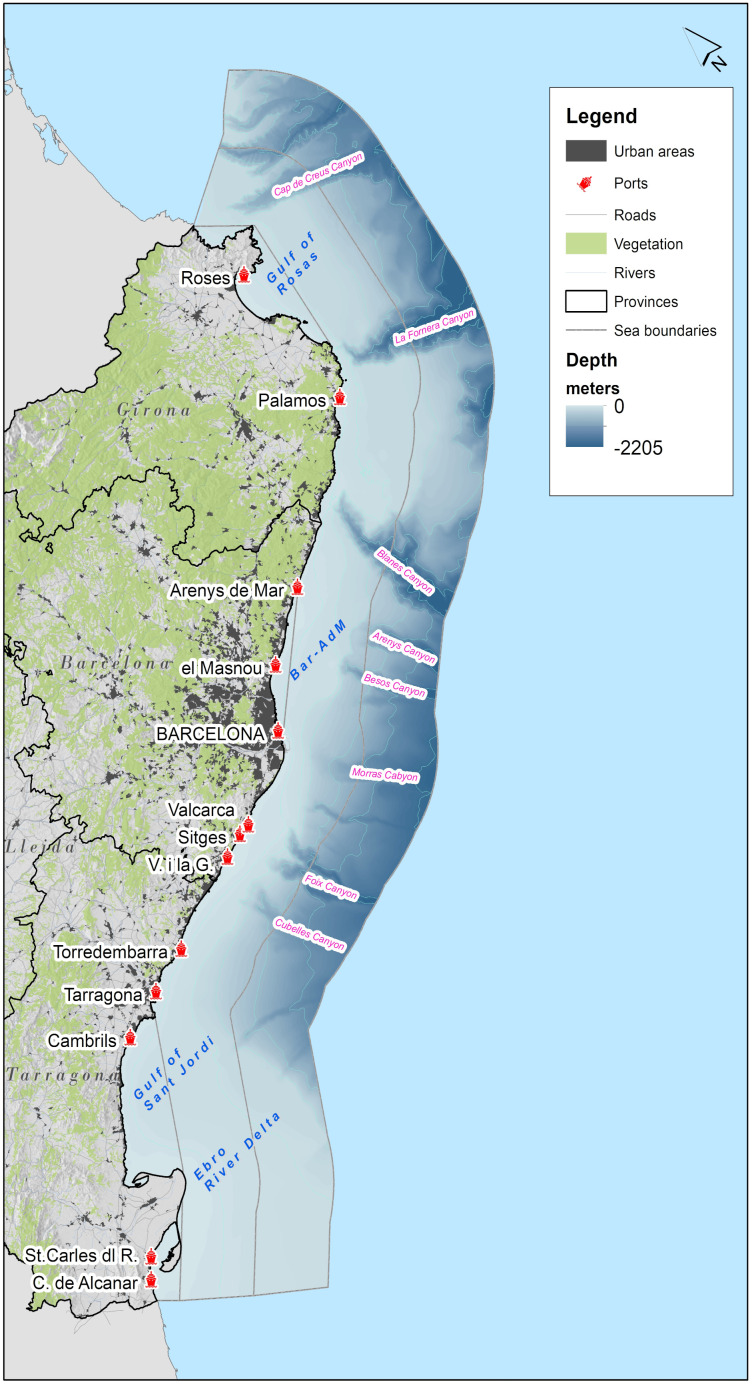
Inland waters, Territorial Waters and Contiguous zone; including bathymetry of the study area and most representative submarine canyons.

The regional study area is located in the Levantine-Balearic subdivision (Western Mediterranean) defined within the *Planes de Ordenación del Espacio Maritime* (POEM), the national maritime spatial plan of Spain ( Figure 2). In order to make a MSP relevant analysis on regional level we define the study area as the regional Internal Waters (IW) including the three subregions, The Gulf of Sant Jordi (1151 km ^2^; 6.3%), the IW of Barcelona-Arenys de Mar (Bar-AdM; 122.8 km ^2^; 0.7%) and the Gulf of Roses (379.3 km ^2^; 2%), the 12 nm limit defined by the Territorial Waters (8124 km ^2^; 44%) and the Contiguous Zone (8627 km ^2^; 47%). Total area of the study area is 18026 km ^2^. The geospatial data resource for the subregions is provided by the EMODNet Geoviewer ( European Marine Observation and Data Network, 2023). The coastline is 580 km long and the sea depth range is 0 to -2205 meters. The subregions are highly relevant for regional MSP and were selected to demonstrate the data-driven approach of the geospatial tools (MUC and StressorShift analysis) in addressing complex interactions of maritime activities in a subregion that agglomerates the majority of current maritime activities and incorporates all the future maritime activities according to the Spanish MSP. From an administrative point of view, there are three coastal provinces (Girona, Barcelona and Tarragona), north-eastern boundary is with France (Occitania region) and south-western regional boundary is with the Valencian Community (Spain).

### 2.2. MSP conditions in the study area

The POEM distinguishes different types of zoning ( BOE-A-2022-21389):

1) *Priority-Use-Areas (PUA)* are sea areas where current activities take place in their designated space, that where not yet carried out. These are sea areas where general-interest must be guaranteed by minimizing the risks to these areas.

2) *High-Potential-Areas (HPA)* are sea areas where certain sectoral activities whose potential development is foreseeable and which require the identification of the most suitable space for its development to minimize potential environmental effects and maximize synergies and coexistence between the different uses and activities ( POEM, 2021). We apply these zoning concepts inside the study area as follows: *i*) *HPA - Offshore Wind Energy* (OWE) refers to high potential areas for renewable energy located in the Territorial Waters in front of the Gulf of Roses. One of the projects described in the *Initial Environmental Assessment* proposals named Tramuntana ( BlueFloat, 2021) foresees the installation of about 63 floating wind turbines (15 MW each; 137 m turbine height). We apply this potential OWE design proposal due to the availability of the *lon/lat* coordinates of the park extent. For other proposals of installation capacity and design in the Gulf of Roses we refer to 4COffshore (2023) Platform. Minimum distance to shore is approx. 14 km (distance to closest port is approx. 24.6 km). *ii*) *PUA – Biodiversity* include protected marine spaces, including the Nature 2000 Network, subject to both national and regional management. *iii) PUA - National Defence* comprise military areas used for National Defence activities in amphibious, underwater and surface military exercises and *iv) PUA - Aggregate Extraction* includes areas containing strategic sand deposits, necessary for coastal protection measures and climate change mitigation. HPA areas include: *v) HPA - Biodiversity* includes expert-based definition of new areas of high ecological value of habitats, birds, cetaceans that according to the POEM (2023) that may be suggested as Natura 2000 Sites to accomplish Biodiversity Strategy 2030 targets and *vi*) *HPA - aquaculture* are new areas for aquaculture facilities development defined by the Junta *Nacional Asesora de Cultivos Marinos* ( JACUMAR, 2023).

### 2.3. MSP settings and geodatabase development

The multi-sectoral MSP database ( Table 1) is fully open source composed by publicly accessible data from international ( AAPG-Explorer, 2019; GFW, 2021; PADI, 2023; Protected Planet, 2023), European ( European Marine Observation and Data Network - EMODNet Geoviewer, 2023; Tools4MSP - Menegon *et al.*, 2018a), national ( BlueFloat, 2021; CEDEX-INFOMAR, 2022; CNMC, 2022; POEM, 2021) and regional ( GenCat, 2022a; GenCat, 2023) data providers. In order to optimize the relevance of the regional study for decision-making and harmonize the modelling procedures we structure the MSP database according to the POEM’s disposition of zoning of maritime activities into current uses and future uses. The database includes 13 existing maritime uses ( *E*) and 4 future uses ( *F*) that are not in place at the current stage (HPA: offshore wind energy and material extraction, aquaculture and biodiversity). The geodatabase development includes three types of indicators, namely intensity of maritime activities; presence/absence of maritime activities and planning prioritization areas (PUA and HPA). In Figure 4 of the results section we provide a synthesis map of the existing and future maritime activities in Catalonia.

**Table 1.** Regional MSP geodatabase: Overview of multi-sectoral geospatial data resources collected for the study area. Note: C – Current uses and F – Future uses. Annex 2 in Depellegrin and Martí Llambrich (2024) provides a visualization of the geodatabase.

Human activity	Status	Description	Source
*PUA*			
Shipping corridors	*C*	[ *trips/cell*] The dataset uses AIS data from the year 2021 including different shipping types of shipping activities Tanker, Cargo, Sailing, Passenger	EMODNet Geoviewer, 2023
Commercial Fishery	*C*	[ *hours/cell*] Hours of fishing by different fishing vessels (Trawling Tuna pursue seines, set_gillnets, pole_and_line, drifting long lines, dredge fishing).	GFW, 2021
Aquaculture	*C*	[ *presence/absence*] Finfish and active mollusc aquaculture sites.	EMODNet Geoviewer, 2023; Gencat, 2022b
Marinas	*C*	[ *number of berths*] 54 marinas of Catalonia including berth numbers.	PdG, 2023
Underwater Cultural Heritage	*C*	[ *presence/absence*] 10 shipwrecks within 60 meter depth accessible for recreational scuba diving	PADI, 2023
Oil and Gas (wells, licensing sites oil&gas platforms)	*C*	[ *presence/absence*] Casablanca Oil Platform, exploration wells and active hydrocarbon exploration areas. The Oil Platform and the wells were prepared for the analysis by applying a safety buffer of 500 meters	EMODNet Geoviewer, 2023; UNCLOS, 1982
Cables & Pipelines	*C*	[ *presence/absence*] Includes actual routes of telecommunication cables. The dataset was prepared by applying a safety buffer of 500m. The oil and gas pipeline connects the Casablanca platform to Tarragona port. A safety buffer of 500 meters was applied.	AAPG-Explorer, 2019; CNMC, 2022; EMODNet Geoviewer, 2023; UNCLOS, 1982; UNCLOS, 1982
Ports	*C*	[ *presence/absence*] 13 regional industrial ports.	EMODNet Geoviewer, 2023
Ports areas	*C*	[ *presence/absence*] 2 anchoring areas in front of Barcelona and Tarragona port.	GFW, 2023
Desalination	*C*	[ *presence/absence*] Desalination plants located in coastal municiaplities only.	EMODNet Geoviewer, 2023
Discharge points	*C*	[ *presence/absence*] Waste water discharge points	EMODNet Geoviewer, 2023
PUA - Nature protection	*C*	[ *presence/absence*] These areas comprise the protected marine spaces, including the Nature 2000 Network, subject to both national and regional management. The regulation of uses and activities within these areas is done by the management body responsible for that protected space, using the relevant space management tool.	Protected Planet, 2023
Aggregates extraction	*F*	[ *presence/absence*] The areas identified in this category contain strategic sand deposits, extraction of which may be necessary for coastal protection measures, including combating climate change	POEM, 2021
National defence	*C*	[ *presence/absence*] The areas identified with this category comprise the areas used for National Defence activities in amphibious, underwater and surface military exercises.	EMODNet Geoviewer, 2023
*High Potential Areas (HPA) ^1^*			
*HPA*			
Offshore Wind Energy (OWE)	*F*	[ *presence/absence*] Areas for possible development of infrastructure for the commercial exploitation of offshore wind energy. Also hybrid renewable energy technologies can be considered. We use the polygon proposed by BlueFloat (2021) for the Tramuntana project. This includes a submarine corridor (sea-land interconnection with OWE) The dataset was prepared by applying a safety buffer of 500m. The possible landing station is also included.	BlueFloat, 2021; EMODNet Geoviewer, 2023; POEM, 2021
Biodiversity	*F*	[ *presence/absence*] Areas with high-potential for benthic habitats, high-value areas for birds and cetaceans, high-value areas for species of community interest and high-value areas for cetaceans.	POEM, 2021
Aquaculture	*F*	[ *presence/absence*] Areas for developing new aquaculture facilities. They comprise those areas provided by the regional authorities, through JACUMAR, in the Proposal for spatial planning of aquaculture. Within the HPA zones of aquaculture development we apply a 5 km buffer from ports to define potential areas for development.	POEM, 2021

### 2.4. Marine protection targets

The developed MSP database is applied to address the 30% sea area protection commitments defined within the Biodiversity Strategy 2030 (BS 2030). The BS2030 is Europe’s comprehensive plan for the conservation and restoration of nature ( EEA, 2023). It is the framework mentioned in the Royal Decree 150/2023 approving the Spanish Maritime Spatial Plan in February 2023. To monitor the 30% protection target in the study area we apply the method developed by the European Environment Information and Observation Network (EIONET; Agnesi *et al.*, 2020). EIONET performs three-annual monitoring of progress in MPA implementation in European seas and is therefore guiding instrument for the monitoring of the 30% protection target. In line with Agnesi *et al.* (2020) and BS2030 we calculate the distance to Biodiversity Strategy Target 2030 ( $BST_{30\%}^{i}$ ) by taking into consideration three types of MPA areas according to the areal percentage of *i*) Natura2000 MPA network (SPA - Special Protected Areas and SIC - Site of Community Interest; *p _SPA/SIC_*), *ii*) national/regional designated areas ( *p _NAT_*) and *iii*) Regional Sea Convention ( *p _RSC_*). The distance refers to the protection coverage in % compared to the 30% target of the BS2030. The dataset used for the calculation is collected from the Protected Planet Database for the study area. The following algorithm is applied for the *i-th* sea space (Inland Waters, Territorial Waters and Contiguous Zone) as follows:


$$BST_{30\%}^{i}=p_{PUA}+p_{HPA}$$

whereas,


$$p_{PUA}=p_{SPA/SIC}+p_{NAT}+p_{RSC}$$

*p _PUA_* are the Priority Use Areas for Biodiversity that reflect the existing marine protected areas in the region (see Figure 4a) three types of marine protected areas defined in EIONET ( Agnesi *et al.*, 2020) and *p _HPA_* are the High Potential Areas for Biodiversity, and reflect the maximum extent of possible future marine protected areas development according to an earlier version of the POEM version of the year 2022, prior to the approved POEM of February 2023 ( *Real Decreto 150/2023*).

### 2.5. Maritime Spatial Conflict analysis

Cross-sectorial spatial conflicts emerge when there is competition for sea space and when potential new sectors pose novel sea space demands. In order to analyse the interaction of human activities we apply a Maritime Use Conflict (MUC) Analysis available in the Tools4MSP modelling framework, an MSP-oriented open source geospatial modeling framework ( Menegon *et al.*, 2018a) applied across Europe (North Sea – Gușatu *et al.*, 2021; Baltic Sea – Depellegrin *et al.*, 2020; Strait of Sicily-Malta – SIMWESTMED, 2018). The modelling framework uses a MUC tool based on the methodology provided by COEXIST ( Gramolini *et al.*, 2010) and has the purpose to locate current sea space conflicts and potential future sea space conflicts emerging from potential OWE development and HPA for biodiversity and aquaculture development. The analysis of sectoral conflicts is an important component when drafting or supporting maritime spatial plans ( BALTICScope, 2017; SPro, 2018) and subject of diverse geospatial applications across European seas ( Coccoli *et al.*, 2018; Menegon *et al.*, 2018b; Pataki & Kitsiou, 2022) and around the globe ( Hou *et al.*, 2022; Roy *et al.*, 2022; Socrate & Verón, 2022). Current cases of application focus on geographic scales of regional, national or seabasin level. The MUC is a fully novel application for the study area. It uses an automated procedure to rasterize all input human activities at 1 km x 1km cell resolution and then categorizes the activities according to five traits: vertical, spatial (horizontal), temporal scale, mobility and location. A three-fold system of rules is used to determine the conflict score using pairwise maritime use vs. use relation. A definition of attributes and rules for MUC application are available in the Table 2.

**Table 2.** Attributes for each maritime use and three-rules overview for the MUC.

Attributes	Rational
Spatial	Describes the spatial characteristics of the maritime use
Temporal	Describes the temporal demand of the maritime use
Vertical	Describes the physical characteristics of the maritime use in the water column
Mobility	Describes the behavior of the maritime use in the sea space
**Rules**	
Verticality	if vertical domain of activity 1 is different from vertical domain of activity 2 and no one of them interests the whole water column then conflict score is equal to 0;
Mobility	If both activities are “mobile” then conflict score is equal to the minimum of temporal domain plus the minimum of spatial domain.
Rule 1 & 2 combined	if Rule1 and Rule2 cannot be applied then the conflict score is equal to the maximum value of temporal domain plus the maximum value of spatial domain.

The following algorithm was applied to the study area as follows ( Menegon *et al.*, 2018b):


$$MUC=\sum_{i=1}^{l}\sum_{j=i+1}^{l}c_{i,j}p(U_{i})p(U_{j})$$

- *c = potential conflict score between use i and j*
- *p(Ui) = presence (1) or absence (0) of the i-th human use in the raster cell (1 km x 1 km)*
- *p(Uj) = presence (1) or absence (0) of the j-th human use in the raster cell (1 km x 1km)*

In order to detect conflict shift areas, referring to sea areas having a net increment of spatial conflicts we define a *MUC _Shift_* as the difference among the conflict of current uses ( *MUC _C_*) and future uses ( *MUC _F_*) as *MUC _Shift_ = MUC _C_ –MUC _F_*. A categorization of current and future uses is provided in Table 1.

### 2.5. Stressor propagation among current and future uses

**Figure 3.**
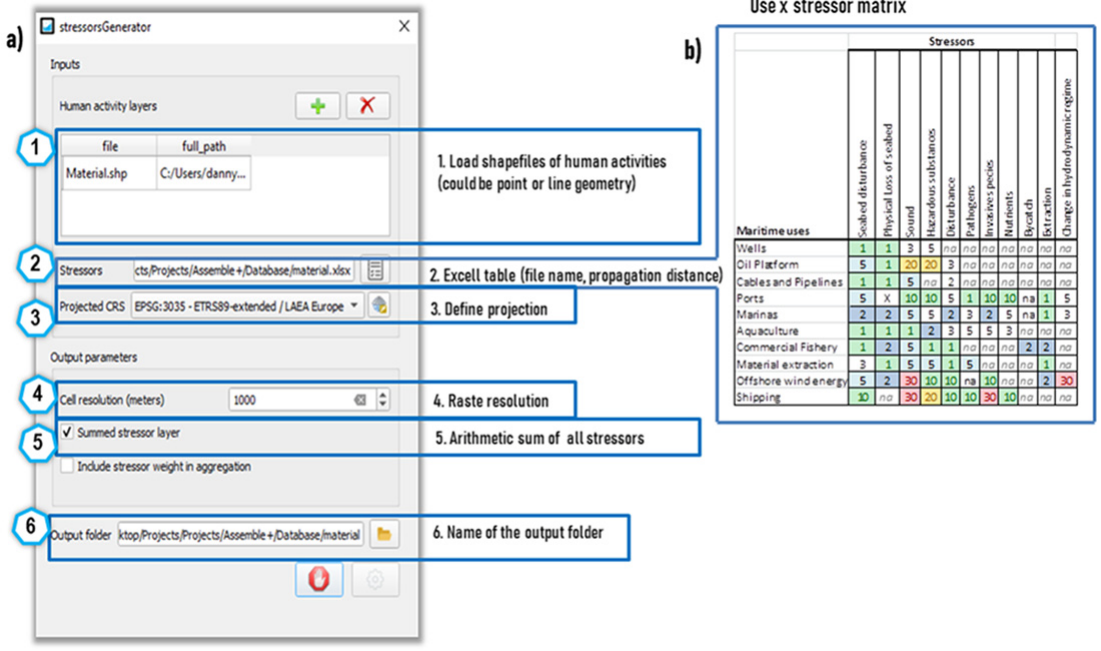
**a**) Graphical-User-Interface, description and steps for setup of the QGIS *StressorGenerator* plugin applied for the study area. **b**) Maritime use – stressor matrix with respective propagation distances in km applied in this study.

To investigate the spatial behaviour of cumulative environmental pressures exerted by existing and future human activities, we perform an exploratory analysis on how the intensity and spatial distribution of stressors shifts with future human activities in the study area could change. For this purpose, we apply a novel QGIS Plugin named *StressorGenerator* that enables the application of an additive multi-stressor propagation model. The model is semi-automated, because final aggregation of the generated stressor rasters for present and future maritime activities is performed by the user through GIS software (e.g. ArcGIS raster calculator) and R-programming language for graphical visualization of results ( R Core Team, 2022). Figure 3 provides an overview of the Graphical-User-Interface of the *StressorGenerator* and the steps to setup a model run: 1) the geospatial layers of maritime activities (in line, point or polygon geometry) are prepared for a study area by converting each layer into a single or multi-point feature and are uploaded in shapefile format; 2) a stressor database in excel table ( *xlsx* format) is developed defining the type of pressure exerted. Currently 11 stressors were adapted from the European Environment Information and Observation Network (EIONET - Korpinen *et al.*, 2019). The propagation of stressors is isotropic using a distance range from *1 km*-local (e.g. seabed disturbance, physical loss of seabed, etc…) stressor to *30 km*-long range stressor (e.g. underwater noise or change of hydrodynamic regime) by different maritime activities. In absence of a dedicated underwater noise propagation model and hydrodynamic model to represent for instance wind wake, the isotropic propagation of the *StressorGenerator* above 30 km is considered too uncertain. The advantage, of the use of EIONET stressor categories is that they were specifically standardized for each European seabasin and therefore enable a comparative use across different marine biogeographic areas; 3) define a common projection for the shapefiles (EPSG-3035); 4) define cell resolution (1 km x 1km); 5) create a raster of the summed stressors for each human activity (resolution 1 km ^2^) and finally 6) define path to store results in an output folder. The stressor model is climate change aware by incorporating the thermal sea water stress as Mediterranean averaged sea-surface-temperature 99th percentile extremes for the period 1987-2019 (32 years) retrieved from Copernicus Marine Service ( CMEMS, 2023). Also, to address eutrophication phenomena we apply a *Chlorophyll-a* anomaly for the years 2006-2021 using Copernicus Climate Service ( CCS, 2023).

**Figure 4.**
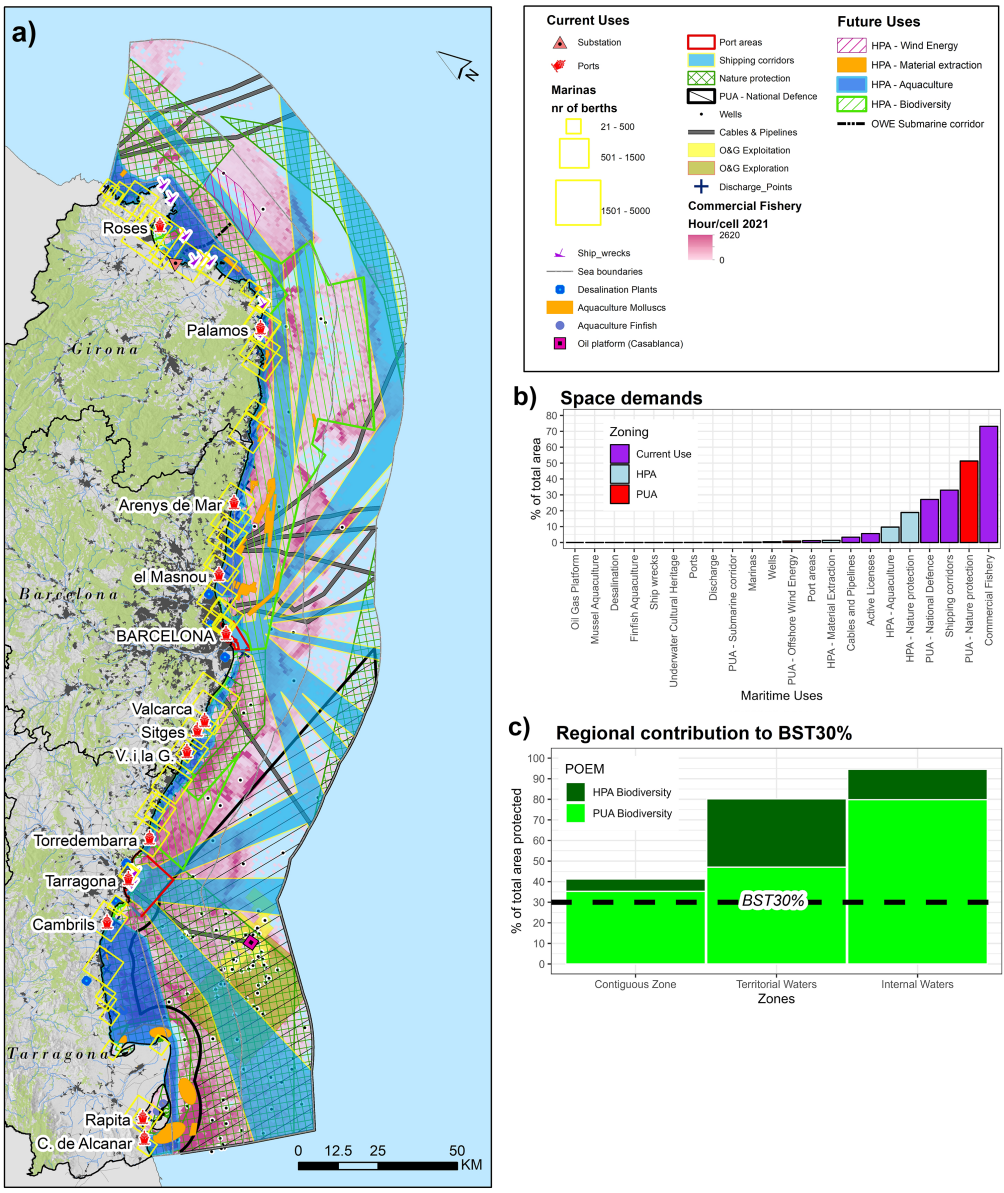
**a**) Spatial distribution of maritime activities with according spatial distribution of current uses and PUA/HPA; **b**) space demands expressed in % of total study area and **c**) barplot, developed in ggplot2 ( R Core Team, 2022) representing regional contribution to 30% sea protection target defined by the Biodiversity Strategy (BST30%) by the three subregion (Internal Waters; Territorial Waters and Contiguous Zone)

The equations below describe the additive nature of the stressor propagation model applied within the *StressorGenerator* for current uses ( $S_{additive}^{c}$ ), future uses ( $S_{additive}^{F}$ ) and current + future uses *:*


$$S_{additive}^{c}=\sum_{k=0}^{n}S_{U_{C}}\;\text{(stressors}\;\text{for}\;\text{current}\;\text{uses),}$$

$$S_{additive}^{F}=\sum_{k=0}^{n}S_{U_{F}}\;\text{(stressors}\;\text{for}\;\text{future}\;\text{uses)}$$

and


$$S_{additive}=S_{additive}^{c}+S_{additive}^{F}\;\text{(summed}\;\text{stressors)}$$

Whereas *S _U _C__* are the stressors exerted by *n* current activities and *S _U _F__* by *n* future uses as defined in Table 1. The model transforms all shapefiles into rasters and then performs a normalized inverse Euclidean distance to produce standardized stressor propagation rasters of value (1-0). The equation for the standardized Euclidean distance for current or future stressors is defined as follows: *D*_*stress*_ = 1 – (( *S*_*U*_*C/F*__ – *S*_*U*_*C/F*__ *min*) / ( *S*_*U*_*C/F*__ *max* – *S*_*U*_*C/F*__ *min*))) * ( *S*_*U*_*C/F*__ *max* – *S*_*U*_*C/F*__ *min*) + *S*_*U*_*C/F*__ *min*.

PUA for biodiversity (including Natura2000 MPA network; national/regional designated areas and Regional Sea Convention) and HPA for biodiversity are omitted from this model because they are considered as stressor receiving sea areas. In order to detect stressor shifts, referring to sea areas having a net increment of stressors we define a *S _Shift_* as the difference among the current pressures ( *p _U _C__*) and future use pressures ( *p _U _F__*), as *S*_*Shift*_ = $S_{additive}^{c}$ – $S_{additive}^{F}.$

## 3. Results

### 3.1. Zoning patterns and sea space demands of current and future sea uses

Figure 4a presents the multi-sectoral distribution of current maritime activities in Catalonia’s regional sea. A fundamental zoning distinction can be noticed: 1) The southern subregion of the regional sea in front of Tarragona Province is highly crowded in its coastal and offshore areas due to the presence of military areas, protected areas, oil and gas sector (Casablanca Platform, pipeline, wells and active licences), finfish and mollusc aquaculture activities (Ebro Delta), intense shipping traffic and port activities. In comparison the northern subregion of the regional sea in front of the Province of Girona, the oil and gas sector is almost absent, there are numerous underwater cultural heritage sites. 2) In the Inland Waters of the Gulf of Sant Jordi and Gulf of Roses intense fishing activities inside their protected areas can be noticed, presence of marinas (about 9170 berths in the Gulf of Roses and 2558 berths in the Gulf of Sant Jordi respectively) and aquaculture facilities. 3) High intensity patterns of commercial fishery concentrate also inside protected areas in the Territorial Waters, and especially in three SPA Birds sites, namely *Espacio marino del Delta de l'Ebre-Illes Columbretes*; *Costes del Garraf/Espacio marino del Baix Llobregat-Garraf* and *Espacio marino de l'Empordà*.

Figure 4b describes the space demand in % to the total study area. Future maritime activities (HPA - Biodiversity, PUA - offshore wind farm with submarine corridor, PUA - Material Extraction sites and HPA - Aquaculture) total an overall increase in sea space demand of 31%. It is worth noticing that the increase of anthropogenic activities in coastal areas and in offshore areas is due to the development of new material extraction sites and the potential development of an offshore wind energy site and the extension of areas of conservation as HPA biodiversity sites.

Commercial fishery covers the most significant amount of space with 73% followed by marine protected areas with 51% respectively ^1^. Shipping corridors cover about 33%, but can be considered to occupy more extensive space when considering smaller intensity shipping corridors especially in coastal areas. Another important sea use is military areas (27%) in the southern subregion of the study area in front of the Ebro Delta. Future maritime activities include a +19% increase of HPA for biodiversity. The net gain of potential regional area-based management features compared to the existing sites is 37% with a resulting potential of 70% of total regional sea space protected in Catalonia. Other future uses are HPA for aquaculture development (+10% of new sea space required), material extraction (+1.4% of new sea space required). Comparably, only 1% of sea space would be required by the HPA for OWE (0.9%) including the interconnector sub-corridor (0.1%). Also worth noting is that 13 maritime activities have sea space demands ≤1% (see Figure 4b).

### 3.2. Marine nature protection targets

In total 51% of the study area is protected, meaning that the BS2030 protection target of 30% is potentially met for this segment of the study area (see Figure 4c). In particular, in the Catalan sea space 80% of Internal Waters, 47% of Territorial Waters and 35% of the Contiguous Zone are marine protected areas. While it is not the scope of this study to discuss the suitability of the HPA - Biodiversity sites as potential protected areas, their integration into some form of area-based management regime would result into about 95% (+15% net gain) protection of Internal Waters, about 80% (+33% net gain) protection of Territorial Waters and about 41% (+6% net gain) protection of the Contiguous Zone. This would make Catalonia one of the most protected regional sea areas in the Mediterranean and Europe. However, where the challenges rely is to include in the 30% target a 10% of strictly protected area. There are three strictly protected areas in the Costa Brava ( *Les Illes Medes; Les Llaunes and Cap de Norfeu*; Protected Planet, 2023). While they are classified as marine protected areas they are spatially land-based strictly protected areas so that the actual contribution to the 10% target of protection of sea space can be considered negligible.

### 3.3. Overview of regional spatial conflicts

**Figure 5.**
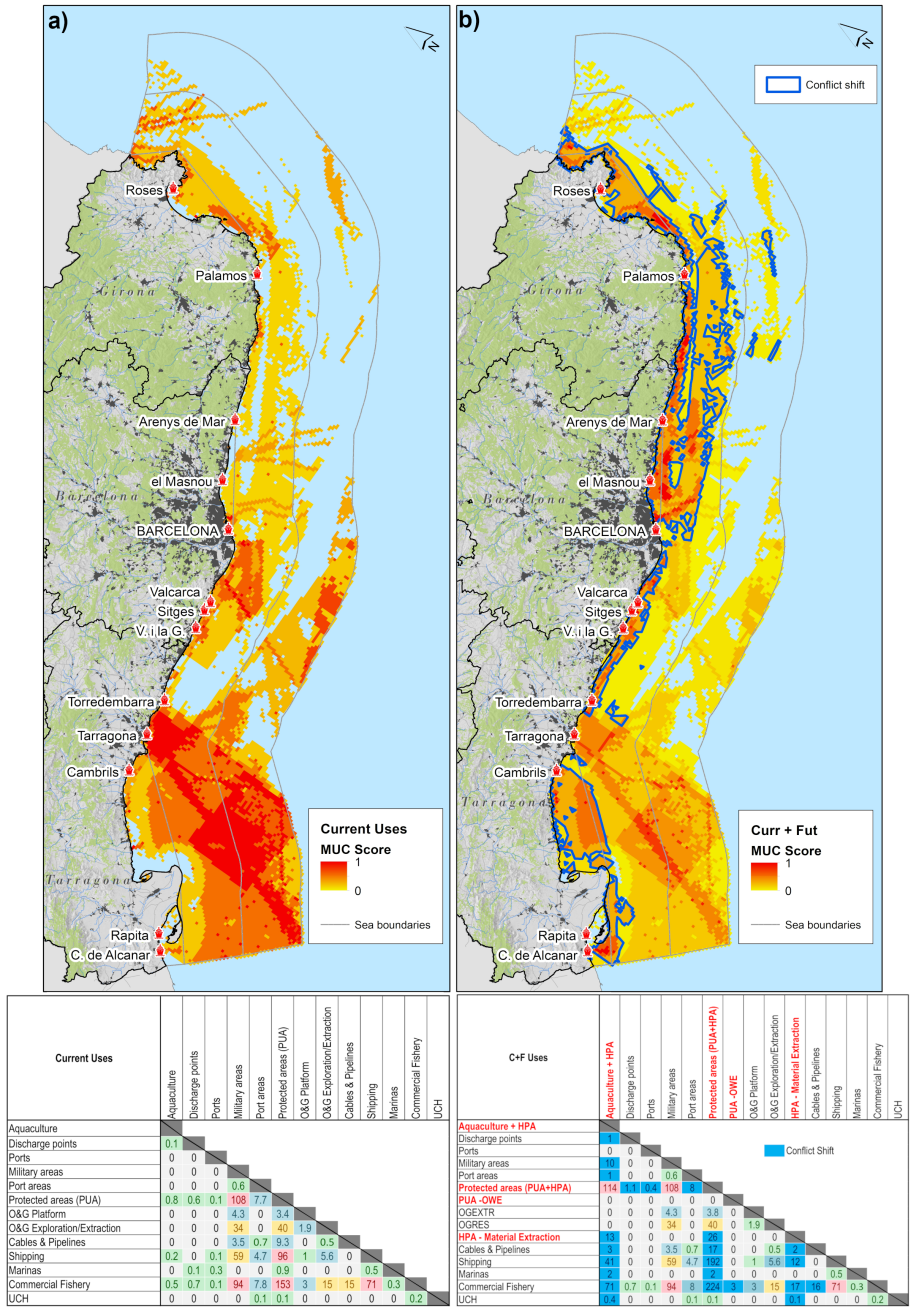
**a**) MUC of current uses; **b**) MUC of current uses and future uses including PUA/HPA zoning; **c**) conflict matrix for current uses **d**) conflict matrix including current and future uses including conflict shift (blue cells), where a net increase of conflict score is expected.

About 56% of the sea space has some degree of spatial conflict. Figure 5a identifies the maritime spatial conflicts of current uses of the sea space. The highest MUC index (score ~1) are located in front of the Ebro river outlet due to the presence of protected areas in combination with intense commercial fishing activities, the presence of the Oil and Gas sector (Casablanca Platform, wells and exploration/exploitation fields), the presence of military areas and shipping corridors departing from the port of Tarragona. On overall, based on the conflict matrix, highest spatial conflict occurs among commercial fishery sector *vs* marine protected areas (score 153), commercial fishery *vs* military areas (score 94) and commercial fishery *vs* shipping (score 71). Other noticeable spatial conflicts occur among military areas *vs* protected areas (score 108) and military areas *vs* shipping (score 59).

The integration of future uses will increase spatial conflicts by 18% in the study area. Figure 5b highlights the emerging future spatial conflicts with presence of future human activities of the sea and conflict shift areas, where net gain of conflict score will occur. Conflicts increase in Inland Waters due substantial potential for HPA - Aquaculture development in the Gulf de Sant Jordi and Gulf of Roses and in coastal areas from Barcelona-Vilanova i la Geltru and from Barcelona-Palamós due to the potential increase of aquaculture development and HPA - material extraction sites. The conflict matrix including future uses shows that, HPA – Aquaculture sites can increase the spatial conflict with commercial fishery and shipping. HPA – Biodiversity sites increase the spatial conflict with commercial fishery, shipping, cables and pipelines and coastal tourism. HPA - Material extraction with aquaculture and protected areas. Surprisingly, the spatial conflicts in the Territorial Waters due to the potential development HPA - OWE remain negligible. This is because compared to other regional sea areas the area has comparably low intensity maritime activities.

### 3.4. Anthropogenic stressors in the Catalan sea space

**Figure 6.**
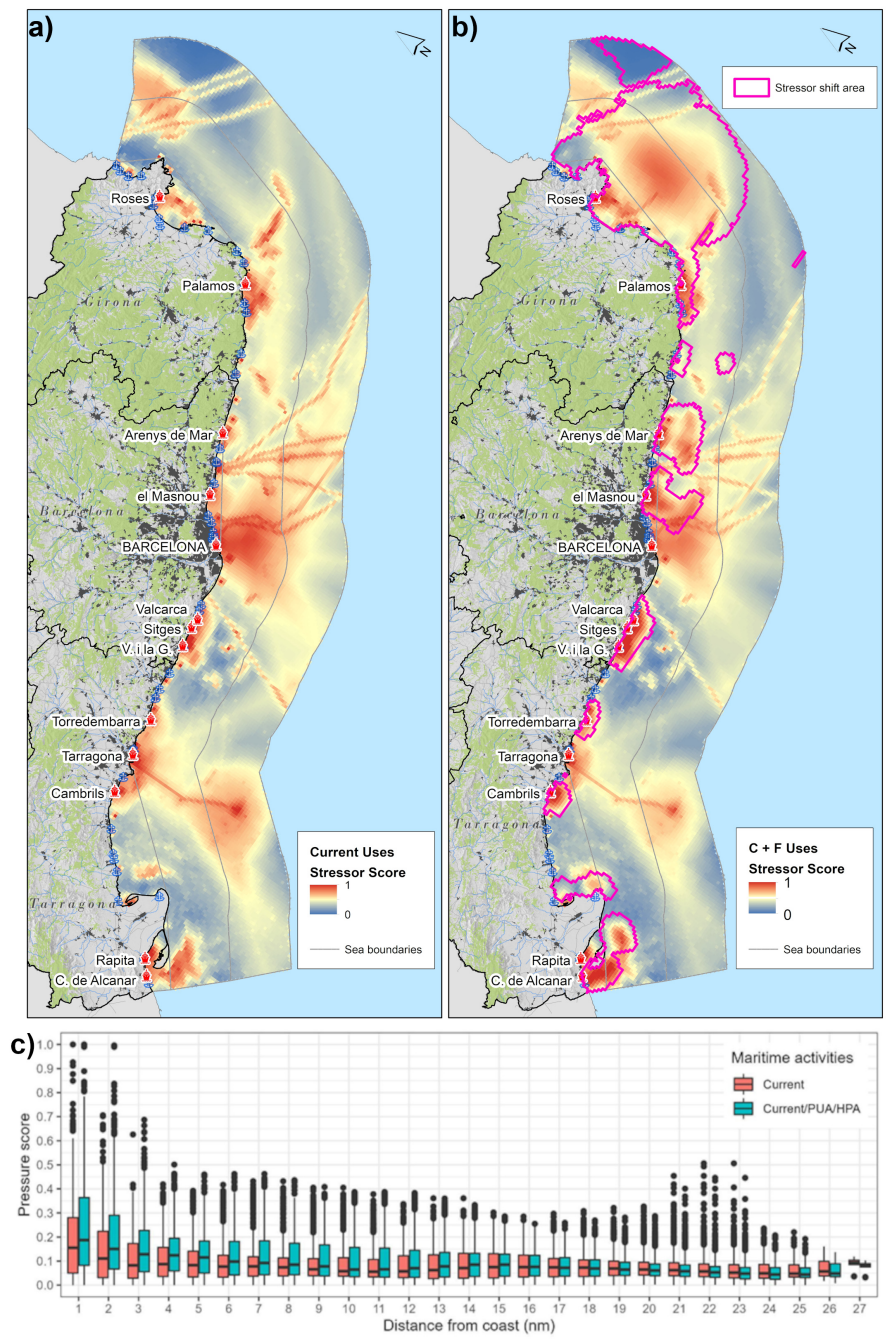
**a**) Stressors exerted by current uses; **b**) additive stressors generated by current and future uses with highlighted areas of stressor shifts; **c**) Boxplots comparing stressor shifts in current *vs* current and future sea uses. Note: dots refer to maximum and minimum outliers.

The Figure 6 presents current anthropogenic stressors and stressor shifts due to future uses. According to model results the sea areas of highest cumulative stressors are located in front of Barcelona, Tarragona and Palamós ports, in the Internal Waters (Gulf of Roses and Sant Jordi) and the entrance to the industrial port, the southern subregion of the Ebro Delta, Gulf of Roses. In the Territorial Waters high stressor areas are caused by intense commercial fishing activities located in front of Cap de Creus, sea areas off Palamós and southern Ebro Delta. In the contiguous zone, stressors are generated by offshore activities, shipping corridors and by the Oil & Gas Platform in front of the Ebro Delta.

The Stressor shift areas in Figure 6b show that cumulative stressors emerging from future sea uses increase in coastal waters, especially in the Barcelona Province and southern Ebro River Delta and Territorial Waters of Girona Province.

Figure 6c describes the current and future stressor shifts as distance gradient from coastline in nautical miles (nm). The 1–4 nm area is subjected to approx. 30% of the stressor intensity. From Figure 6 (right) it can be noticed that the sea areas subjected to stressor shift are either localized at subregion scale, while other sea areas are affected by stressor shift in multiple subregions, such as for instance in the Gulf of Rosas and the adjacent Territorial Waters.

## 4. Discussion

The research presents a database and state-of-the-art analytical techniques to support decision-making in MSP exemplified for the coastal region of Catalonia. An exploratory analysis resulted to be particularly useful in this context, because it informs decision-makers about the pre-existing sectoral conditions and forecasts emerging trends from future maritime activities based on the PUA/HPA zoning mechanisms defined within the national POEM. The presented models can be deployed at different spatial scales (from national to seabasin level). In the following section we formulate five challenges emerging from the analysis that reflect the horizontal multi-sectoral objectives defined in the Spanish Maritime Spatial plan. Challenge 1 & 2 addresses co-existence among existing maritime activities and emerging ones (HMO-2); challenge 3 discusses minimization of spatial conflicts (HMO-1); challenge 4 discusses the need for coordination among administrations in coastal-marine interface and challenge 5 discusses the need for further scientific research, by highlighting data gaps and model limitations.

### 4.1. Challenge 1: Marine protection beyond percentage

Marine protection and enforcement of conservation objectives remains a main challenge in the Western Mediterranean and in the study area ( Company *et al.*, 2012; García *et al.*, 2021; Perry *et al.*, 2022). Our analysis shows spatial conflict of protected areas with various maritime activities in the region, especially commercial fishery, coastal tourism (in the form of marinas), shipping and oil and gas extraction and exploration ( Figure 5). Recent scientific literature and Mediterranean initiatives evidence that marine protected areas suffer from increased anthropogenic pressures from existing maritime activities in the study area especially from high-risk ^2^ commercial fishing activities (e.g. trawling) in protected areas of the Western Mediterranean ( Mazaris *et al.*, 2019; MedReAct, 2022; Med Sea Alliance, 2023; Muñoz *et al.*, 2018; Perry *et al.*, 2022), in particular in SIC - *Sistema de cañones submarinos occidentales del Golfo de León* ( García *et al.*, 2021), in submarine ecological hotspots such as *La Fonera Canyon* on the Catalan margin of the NW Mediterranean ( Martín *et al.*, 2014; Paradis *et al.*, 2017). Our results (see map Annex 3 in Depellegrin & Martí Llambrich, 2024) indicate that about 56% of the fishing efforts (including trawling) in 2021 in the regional sea of Catalonia occur inside the actual marine protected areas (PUA - Biodiversity) and another 26% could potentially occur in potential future HPA – Biodiversity sites. In addition, environmental pressures caused by coastal and nautical tourism are posing continuous threats to coastal areas and vulnerable habitats ( Gonzalez *et al.*, 2020). Significant is that about 62% (20,037 berths out of 31,000 total marina berths; PdG, 2023) are located inside or in front of marine protected areas: *i*) 57% (11,434 berths) in marinas of Girona Province; *ii*) 19% (about 3,805 berths) in marinas of Barcelona Province and *iii*) 24% (about 4,798 berths) in Tarragona Province (see map of Annex 4 in Depellegrin & Martí Llambrich, 2024). In late December 2022 the Spanish Government approved the national strategic plan for natural heritage and biodiversity ( Miteco, 2022a). A key action point of the plan is the establishment of 8 new marine Natura2000 sites in the Spanish sea space within 2023–24 in support of the EU Biodiversity Strategy 2030 requirement to protect 30% of the sea areas ( Miteco, 2022a; see map Annex 5 in Depellegrin & Martí Llambrich 2024). A key aspect in integrating new MPA is a comprehensive understanding of the MSP conditions and maritime activities occurring in the proximity of candidate MPA areas ( Grorud-Colvert *et al.*, 2021).

Our results provide practical insights that can support new marine protected areas selection:

1) It is of pivotal importance to reach the 10% target of strictly protected areas ideally in the 1–4 nm conflict zone ( Figure 5b) that can be *de facto* be no-go-area. This would relieve ecological resources from current spatial conflicts and anthropogenic stressors highlighted in this study.
2) Evaluate sea areas allocated as future HPA - Biodiversity areas settled in the POEM as candidate areas of the Natura2000 for regional proposals.
3) Align the candidate marine Natura2000 network selection with the Spanish Green Infrastructure Strategy (Article 15.3 of Law 33/2015; Biodiversity-Europe, 2023) by considering offshore areas like submarine canyon protection due to their high ecological functions and connectivity role ( Fernandez-Arcaya *et al.*, 2017; Paradis *et al.*, 2017).
4) Couple the practical insights of the study based on data-driven approach and geospatial techniques with participatory MPA co-design process ( Horta e Costa *et al.*, 2022) and the analysis of anthropogenic effects as a function of different levels of protection of existing MPAs and future Biodiversity protection sites.

### 4.2. Challenge 2: Offshore Wind Energy, a new regional sea space demand

In European seas, the development of OWE is a strategic cornerstone of the transition towards a sustainable Blue Economy ( EC, 2020; EC, 2021). In 2022, The Spanish Ministry of Ecological Transition has defined a national roadmap for offshore wind and marine energy development ( Miteco, 2022b), setting the pathway for an industrial and technological transition using ocean renewable energy development as a vector of innovation. The presented study provides a set of valuable insights in relation to the potential OWE development site worth to be highlighted:

1) The potential OWE site in front of the Gulf of Roses has low to negligible (score 0 to 0.3; Figure 6d) multi-sectoral spatial conflicts. AIS-tracked maritime transport activities have relatively low route intensity compared to intensive shipping activities in other areas from Barcelona and Tarragona port to Balearic Islands, Morocco and Italy. Fishery displacement that is one of the most severe socio-economic impacts of potential OWE farm development ( Gill *et al.*, 2020; Marine Scotland, 2022; MSP-EC, 2021), show that the fishing effort in area occupied by the farm stays relatively low, because part of OWE site is partially coinciding with a fishery closed-area ( CSIC, 2022).
2) The MUC analysis ( Figure 5d) evidences that potential OWE site is localized outside three types of commonly constrained areas: military areas ( Abramic *et al.*, 2021; Díaz *et al.*, 2019; Sourianos *et al.*, 2017), marine protected areas ( POEM, 2021) and high intensity shipping lanes ( Argin *et al.*, 2019; Peters *et al.*, 2020; Virtanen *et al.*, 2022).
3) According to the Global Wind Energy Council dataset ( GWEC, 2021), about 30% (5497 km ^2^) of the study area has a potential suitable sea space for floating OWE development (see Annex 6 OWE potential *versus* constrains map in Depellegrin & Martí Llambrich, 2024). This suitable space is distributed over the northern subregion (Gulf of Roses) and the southern subregion (Tarragona – Ebro river outlet). The collected regional MSP data and model results show that the Tarragona-Ebro river outlet subregion is a highly constrained area, because *i*) it is located inside the SPA- *Espacio marino del Delta de l'Ebre-Illes Columbretes*, *ii*) it is an area for national defence, *iii*) it is a sea area of high intensity maritime traffic (including tankers cargos and ferries) and of intense fishery activity (see MUC score Figure 5a).

### 4.3. Challenge 3: Crowded coastal areas

Model results suggest that zoning types defined within PUA and the HPA are likely to increase potential spatial conflicts and pressure to coastal areas especially in the coastal areas from 1–4 nautical miles ( Figure 6c). While the scope of the study is not to address the effects of the stressors exerted by the multiple maritime activities on ecological resources, it is intuitive that the additive nature of the stressor model will localize highest ecological impacts as a function of the geographic co-occurrence of the maritime activities, eutrophication phenomena and thermal stress from climate change. As a consequence, an emerging solution to tackle current and future sea space competition and alleviate coastal areas from the additive anthropogenic stressors in the 1–4 nm zone, is for example a regional-scale analysis of opportunities for ocean multi-use ( Schupp *et al.*, 2019). Examples of formal integration of multi-use or co-location solutions are included into very recent marine governance frameworks, such as for example the Portuguese Maritime Spatial Plan ( Calado *et al.*, 2024; PSOEM, 2023), pilot MSP studies such as for Emilia-Romagna ( Barbanti & Perini, 2019), national scientific research projects ( ACUFLOT, 2022) and other studies ( EU-MSP, 2023; Weiss *et al.*, 2018). For Catalonia, regional multi-use planning measures would be effective at stimulating a move of sea space use outside the 4 nautical miles coastal area by current sea uses (coastal tourism, fishery and aquaculture). Positive regional multi-use examples combining coastal tourism with tuna aquaculture exist in L’Ametlla del Mar in Tarragona Province ( TripKay, 2023), while ongoing research for addressing the sustainability of OWE potentials with aquaculture development were addressed in the Aquawind project ( https://aquawind.eu/) in the Canary Islands. In highly crowded regions, such as inland waters, the analysis of spatial interactions for certain indicators may be overly simplistic. A resolution of 1 square km might prove insufficient for densely populated coastal areas, where activities often occur very precisely and at finer scales. In such scenarios, an effective enhancement could involve adopting a multi-resolution approach or considering a more adaptable spatial interaction model. This could entail not only accounting for overlapping uses but also incorporating buffering techniques or accounting for remote interactions. This may also entail the need to collect more detailed and higher resolution data for those areas at sub-regional level.

### 4.4. Challenge 4: Multi-level governance

In MSP the development of multi-level governance mechanisms of continuous coordination and cooperation among administrations at different territorial scales is essential when addressing ocean sustainability goals. For instance, according to the Spanish Constitution (art. 149) central governments have numerous competences on the sea (such as on offshore energy, national navigation, fishing beyond internal waters, ports of general interest –such as Barcelona and Tarragona ports, according to Annex I.9 of the State Ports and Merchant Navy Law–, defense and Armed Forces), regional governments have responsibilities on coastal and nearshore maritime activities (fishing in internal waters, aquaculture, coastal planning and management, recreational fishing, marinas, tourism, leisure and maritime-coastal sports; art. 148 Spanish Constitution; arts 119&149 of the Statute of Autonomy of Catalonia). Given the complexity of interactions of these sectors, the strengthening of participatory processes and methods with stakeholders at different territorial levels remains pivotal for future iterations of the POEM. Of particular importance are areas of Land-Sea Interaction (LSI). Coastal-maritime areas are highlighted as crowded coastal areas where also potential different administrative structures and responsibilities overlap. For instance regional coastal management plans with the maritime-terrestrial public domain. Above all, a synergic coordination of maritime-coastal governance gains prominence in the light of regional climate change phenomena. Along the national and regional Climate Change Adaptation Strategies ( ESCACC, 2021; Miteco, 2020) an increasing segment of literature pertinent to the Catalan sea areas project intensifying climate-induced threats to the Catalan sea areas, such as 1) reduced habitat availability for pelagic fish due to the synergic effect of climate change and fishery activities ( Ouled-Cheikh *et al.*, 2022); 2) increased high intensity ocean warming events also called Marine Heat Waves ( Hobday *et al.*, 2016) that affect marine biological processes with severe consequences on regional coastal tourism ( Juza *et al.*, 2022) and finfish aquaculture production ( López Mengual *et al.*, 2021) and 3) sea level rise-induced coastal erosion will affect 70% of Catalonia’s beaches in the coming decades ( Cads, 2017). The phenomenon is associated with economic damages to the regional coastal tourism sector estimated at 2.2 billion € (20% of the regional GDP) by the end of the century ( Garola *et al.*, 2022; Jiménez *et al.*, 2017). Our MSP database is climate change-aware, meaning that incorporates a 32 year sea surface temperature anomaly dataset ( CMEMS, 2023), but given the severity and socio-economic importance of the regional coastal environment further datasets should be incorporated to address and project climate change impacts for regional blue economy activities.

### 4.5. Challenge 5: Regional MSP knowledge base and gaps

In terms of sectoral datasets, it is essential to retrieve data that can address spatio-temporal mobility patterns of the tourism sector at a regionally relevant planning scale. In Catalonia this sector plays a prominent role, however datasets are missing that are capable of better interpreting its interaction with other maritime activities and marine protected areas. This gap could be tackled through the use of high resolution remote sensing techniques to detect small boat nautical tourism mobility ( González *et al.*, 2020) and an extension of regional surveys of mooring activities.

Regional datasets of fishing activities by gear type from regional authorities can further complement the datasets ( ICATMAR, 2021) Also datasets on artisanal fishery, which is an important Blue Economy activity for regional coastal communities ( Carbonell, 2020) needs to be further integrated into the analysis. Both datasets would provide additional means for the analysis of spatial conflicts and stressor analysis.

In the study area, the aquaculture development shows to be the Blue Economy sector to have the highest space demands in the Inland waters of the Gulf of San Jordi, the Gulf of Roses and in southern and northern coastal waters of Barcelona ( Figure 4). Given the increasing importance of aquaculture in the Mediterranean ( UfM, 2021) and its potential regional space demands in Catalonia, there is the need to identify MSP-based techniques that enable to better define suitable sites taking into consideration spatial, ecological and socio-economic conditions ( Graham *et al.*, 2020; Porporato *et al.*, 2020; Venier *et al.*, 2021) and define planning strategies that can address the regional ocean multi-use potential.

A further knowledge gap is the absence of regional data derived from local spatial knowledge from maritime sectors. This include socio-ecological knowledge on marine ecosystem services, spawning grounds, fishery closure areas, coastal landscape features and information on regional diving hot-spots, their frequency of visitation or information on recreational mooring sites. This would produce knowledge that could better characterize the regional sea space and act as enabler for more informed spatial planning, stimulate stakeholder participation and increase acceptance of Maritime Spatial Plans ( Pennino *et al.*, 2021; Said & Trouillet, 2020). The regional scope of the study makes it particularly suitable to extend the developed database with the mentioned socio-ecological knowledge.

The stressor propagation analysis developed using a QGIS plugin resulted in a promising instrument to address future planning challenges. For instance, the stressor propagation can be coupled with ecological datasets such as EUNIS Habitat maps ( EU-Seamap, 2021) and different techniques of marine species suitability models ( El-Gabbas *et al.*, 2021; Gușatu *et al.*, 2021; Schickele *et al.*, 2020) in order to perform a risk-based cumulative effect assessment. The *StressorGenerator* can be further complemented with pressure types aligned with MSFD pressures, that offers a more detailed definition of pressures that is commonly used in European cumulative effect assessment studies, such as marine litter, synthetic or non-synthetic compounds, etc… ( Casimiro *et al.*, 2021; Korpinen & Andersen, 2016; Menegon *et al.*, 2023). Also pressure specific spatial models should be integrated, such as simulations of wind turbine wake behaviour (e.g. Raghukumar *et al.*, 2022), hydrodynamic models to better take into account riverine inputs and their 3-dimensional dispersion. This is essential considering the presence of the Ebro river Delta in southern subregion of the study area that can be a source of multiple environmental stressors (micro plastics - Simon-Sánchez *et al.*, 2019; pesticides - Peris *et al.*, 2022; and eutrophication - Busch *et al.*, 2016; Garnier *et al.*, 2021). An improvement within the spatial models proposed in the stressor shift model is the use of open source software package OpenDrift ( Dagestad *et al.*, 2018) programmed in python. The software enables particle drift modelling in marine environments and can for instance be taken into consideration for different pollutant dispersion as a function of depth, such as oil spills.

## 5. Conclusion

The presented study is the first MSP-oriented regional study for Catalonia, addressing the interaction of multiple maritime activities on the marine environment and identifying sea areas of highest stress due to current and emerging future space demands. The results of the research can inform national and regional decision making on the current and future maritime spatial settings of the region of Catalonia. The results provided by this study can be transferred to other coastal regions, on a POEM-subdivision scale (e.g. Levantine-Balearic) or even on the entire national sea space. The presented geo-statistical information provides a valuable starting point to create a regional MSP knowledge database for Catalonia. It is structured to mimic POEM’s maritime sectors and zoning types PUA/HPA and the maritime sectors identified in the regional Maritime Strategy 2030. Ideally, the presented database and models could feed already existing regional GIS-based platforms (e.g. *visor maritim* of the Generalitat de Cataluña: https://sig.gencat.cat/visors/visor_maritim.html) or contribute to future advancements aiming at increasing the regional knowledge on ocean planning opportunities and challenges.

In terms of data, our study highlighted shortcomings on nautical tourism mobility data, commercial/artisanal fishing and high resolved regional ecological dataset for the marine environment that are essential for future regional cumulative effect assessment. In parallel, shortcomings should be tackled on the development of new methods and framework that can extend the traditional MSP paradigm towards more equitable integration of regional marine ecosystem services studies, transition-based science and socio-economic cost-benefits analysis of Blue Economy sectors. Within the progress of the development of future Maritime Spatial Plans, offshore sea areas requiring shipping corridors re-routing or where displacement risks for coastal tourism activities and commercial fisheries are likely should become integral part of SEA through a i) protocol of analysis, ii) data requirements to address the conflicts/synergies and a iii) portfolio of applicable measures.

Our results show that spatial conflicts and stressors occur independently from the territorial administrative structures and responsibilities in coastal-maritime realms. The research provides the seeds for future updates of regional-national adaptation plans and for more integrated governance processes aimed at achieving common ICZM-MSP goals.

## Data Availability

The data that support the findings of this study are derived from the following resources available in the public domain: EMODNet Geoviewer, 2023 ( https://emodnet.ec.europa.eu/geoviewer/); Global Fish Watch, 2021 ( https://globalfishingwatch.org/); Gencat, 2022a ( https://agricultura.gencat.cat/ca/ambits/pesca/proteccio-recursos-litoral/esculls-artificials/llistat-ubicacio/); PdG, 2023 ( https://ports.gencat.cat/mapa-i-sistema-portuari-catala/#puntZonaNord); PADI, 2023 ( https://www.padi.com/); Protected Planet, 2023 ( https://www.protectedplanet.net/en/thematic-areas/wdpa?tab=WDPA); POEM, 2021 ( https://www.miteco.gob.es/es/costas/temas/proteccion-medio-marino/spanishmsplanssummary_tcm30-532936.pdf). Zenodo: Dataset used in Addressing ocean planning challenges in a highly crowded sea space: a case study for the regional sea of Catalonia (Western Mediterranean), https://doi.org/10.5281/zenodo.10263416 Depellegrin and Martí Llambrich (2023). Zenodo: Supplementary material for the manuscript Depellegrin *et al.*, 2024: Addressing ocean planning challenges in a highly crowded sea space: a case study for the regional sea of Catalonia (Western Mediterranean) https://doi.org/10.5281/zenodo.10540658 ( Depellegrin & Martí Llambrich, 2024). The annex 1 to 6 mentioned in this manuscript can be downloaded as Supplementary Material. Data are available under the terms of the Creative Commons Attribution 4.0 International license (CC-BY 4.0).

## Footnotes

^1^ depending on calculation method this could result into 44% (communication from former reviewer).

^2^ According to Perry *et al.* (2022) high-risk refers to fishing gear known to pose potential risk to habitats, e.g. trawling; “High-risk” is the term used by the “EU Action Plan: Protecting and restoring marine ecosystems for sustainable and resilient fisheries” (COM(2023) 102 final; https://oceans-and-fisheries.ec.europa.eu/system/files/2023-02/COM-2023-102_en.pdf)
