# Supplementary material for: Addressing ocean planning challenges in a highly crowded sea space: a case study for the regional sea of Catalonia (Western Mediterranean)
Source: Open Res Eur. 2024 Mar 1;4:46. [Version 1] doi: 10.12688/openreseurope.16836.1 (PMC11222783; doi:10.12688/openreseurope.16836.1)
Supplement: Peer review history

# Reviewer report: Reviewer response for version 1

**DOI:** 10.21956/openreseurope.18191.r41152

**Published:** 2024-07-03

**Authors:** Aurelija Armoskaite

**Affiliations:** 1 Latvian institute of Aquatic Ecology, Riga, Latvia

This paper introduces a methodology for analysis to support maritime spatial planning. This methodology is demonstrated in the Catalonian region, showcasing its value. The paper is interesting, well-written and a timely, valuable contribution to the field of geospatial decision support tools and analysis techniques.  

 The introduction and methods sections provide a thorough overview of the planning region in focus. The introduction describes the missing component of explorative analysis and an evidence base for MSP in a region important for various maritime activities. The methods section then dives deeper into the MSP targets, open geodata availability and the chosen methods. The methods section is clear, transparent, and easily understandable for the readers.

 The results section is structured according to the research objectives clearly outlined in the introduction. The results are illustrated with appropriate and well-designed figures. The results presented are insightful. They reveal current spatial conflicts and stressors and potential shifts in the future, as well as a high regional contribution towards the BST30% targets whilst raising the challenges of designating 10% for strict protection.

 The discussion is engaging and well-rounded. The authors unpack the results thoroughly and discuss them in light of challenges applicable to the study region and beyond.  They offer a range of suggestions on how the results and methods presented in the study can inform the decision-making process and further research.

 Here are some suggestions for the authors to consider:

- The abstract states: 'Potential new aquaculture sites ARE causing…’ I wonder if the present tense is appropriate here, as the focus is on future activities. Consider rewording or perhaps even mentioning the results of conflict and stressor shift analyses.
- Check the resolution of the figures and grammar throughout the text. For instance, there is a missing comma in the second sentence of section 2. Methods: ‘In summary, this includes:…’. There are also a few missing articles (e.g. ‘…THE highest conflicts…’ in the abstract and ‘of pivotal importance now and in THE future to perform…’ in the introduction).
- Consider the downsides of additive stressor/cumulative impact analysis in the discussion.

Is the study design appropriate and does the work have academic merit?

Yes

Is the work clearly and accurately presented and does it cite the current literature?

Yes

If applicable, is the statistical analysis and its interpretation appropriate?

Not applicable

Are all the source data underlying the results available to ensure full reproducibility?

Yes

Are the conclusions drawn adequately supported by the results?

Yes

Are sufficient details of methods and analysis provided to allow replication by others?

Yes

Reviewer Expertise:

Marine spatial planning and ecosystem-based management; social-ecological systems analysis; environmental impacts assessments; geographic information systems and quantitative & qualitative social research methods.

I confirm that I have read this submission and believe that I have an appropriate level of expertise to confirm that it is of an acceptable scientific standard.

# Reviewer report: Reviewer response for version 1

**DOI:** 10.21956/openreseurope.18191.r39952

**Published:** 2024-05-17

**Authors:** Humood Naser

**Affiliations:** 1 University of Bahrain, Sakhir, Southern Governorate, Bahrain

The paper entitled ‘Addressing ocean planning challenges in a highly crowded sea space: a case study for the regional sea of Catalonia (Western Mediterranean)’ provides a case study of applying marine spatial planning. It focuses on regional sea of Catalonia, Spain. Marine spatial planning is considered as an important tool to inform decision making processes related to the use and conflict of the marine environment. Therefore, the current paper topic is important and can provide insights on the approach, methodology and applicability of marine spatial planning, especially in areas with intensive use such as the Western Mediterranean area.

 The methodological approach is scientifically sound. It starts by providing a conceptual framework to highlight the overall approach and the main steps. This can provide the reader with a visual holistic view of the methods used in the study. One important thing in the method is the use of publicly available data. This can contribute to the utilization and application of the available data in marine spatial planning. The method section details the study area location and the current users, geodatabase development, marine protection targets and conflict analysis, and current and future users’ analysis.

 The results section generated maps of the spatial distribution of marine activities of the current and future uses of the area. The discussion provides an account for the main five challenges associated with practical insights. These insights could be very useful for planning and implementation of marine spatial planning as they are focused and based on the outcomes of the study findings.

 Further, there are few notes that might be needed to be addressed:

 Page 3: The last paragraph of the introduction: [ Based on the results ….]. I assume this part could be with the result section, as it specifies five challenges etc. or it might be altered into an objective.

 The resolution of the figures in the online versions are fine, but please double check on the printed version of the paper.

 Page 5 (Ca. Line 3): Please check Nature 2000 0r Natura 2000?

 Page 5 (The last paragraph): What is it meant by: (The MUC is a fully novel application)? Is it developed by the study or utilized, because it is mentioned it is available in the Tools4MSP. The same for page 8 (we apply a novel QGIS plugin)?

 Page 11: please check the use of the word (Surprisingly): because the outcome is negligible and that is due to the low intensity in comparison with other areas.

Is the study design appropriate and does the work have academic merit?

Yes

Is the work clearly and accurately presented and does it cite the current literature?

Yes

If applicable, is the statistical analysis and its interpretation appropriate?

Not applicable

Are all the source data underlying the results available to ensure full reproducibility?

Yes

Are the conclusions drawn adequately supported by the results?

Yes

Are sufficient details of methods and analysis provided to allow replication by others?

Yes

Reviewer Expertise:

NA

I confirm that I have read this submission and believe that I have an appropriate level of expertise to confirm that it is of an acceptable scientific standard.
